# Deciphering the Pharmacological Mechanism of the Herb *Radix Ophiopogonis* in the Treatment of Nasopharyngeal Carcinoma by Integrating iTRAQ-Coupled 2-D LC-MS/MS Analysis and Network Investigation

**DOI:** 10.3389/fphar.2019.00253

**Published:** 2019-03-18

**Authors:** Xuesong Feng, Hailong Shi, Xu Chao, Fei Zhao, Liang Song, Minhui Wei, Hong Zhang

**Affiliations:** ^1^Medical Experiment Center, Shaanxi University of Chinese Medicine, Xianyang, China; ^2^Basic Medical Academy, Shaanxi University of Chinese Medicine, Xianyang, China

**Keywords:** traditional Chinese medicine, *Radix Ophiopogonis*, nasopharyngeal carcinoma, iTRAQ, proteomics, network pharmacology

## Abstract

The herb *Radix Ophiopogonis* (RO) has been used effectively to treat nasopharyngeal carcinoma (NPC) as an adjunctive therapy. Due to the complexity of the traditional Chinese herbs, the pharmacological mechanism of RO’s action on NPC remains unclear. To address this problem, an integrative approach bridging proteome experiments with bioinformatics prediction was employed. First, differentially expressed protein profile from NPC serum samples was established using isobaric tag for relative and absolute quantification (iTRAQ) coupled 2-D liquid chromatography (LC)-MS/MS analysis. Second, the RO putative targets were predicted using Traditional Chinese Medicines Integrated Database and known therapeutic targets of NPC were collected from Drugbank and OMIM databases. Then, a network between RO putative targets and NPC known therapeutic targets was constructed. Third, based on pathways enrichment analysis, an integrative network was constructed using DAVID and STRING database in order to identify potential candidate targets of RO against NPC. As a result, we identified 13 differentially expressed proteins from clinical experiments compared with the healthy control. And by bioinformatics investigation, 12 putative targets of RO were selected. Upon interactions between experimental and predicted candidate targets, we identified three key candidate targets of RO against NPC: VEGFA, TP53, and HSPA8, by calculating the nodes’ topological features. In conclusion, this integrative pharmacology-based analysis revealed the anti-NPC effects of RO might be related to its regulatory impact via the PI3K-AKT signaling pathway, the Wnt signaling pathway, and the cAMP signaling pathway by targeting VEGFA, TP53, and HSPA8. The findings of potential key targets may provide new clues for NPC’s treatments with the RO adjunctive therapy.

## Introduction

Nasopharyngeal carcinoma (NPC) is one of the most common cancers in Southeast Asia, Southern China, the Arctic, northern Africa, and the Middle East (Mohammed et al., 2017). It occurs less frequently in the Western world (McDermott et al., 2001; Lo and Huang, 2002; Parkin et al., 2005). Male patients are more likely to develop NPC and their occurrence rate is nearly twice as high as that in female individuals. NPC starts to develop when tumor cells arise from the epithelium of the nasopharynx. The symptoms of NPC is vague, including lump in the neck, double vision, ear infections, face pain or numbness, headache, hearing loss, difficulty in opening mouth, nosebleeds, stuffy nose, sore throat, and so on (Mohammed et al., 2017). The current treatments of NPC are radiation therapy and chemotherapy to kill cancer cells and stop them from growth and metastasis. There are short-term and long-term side effects of radiotherapy and chemotherapy including nasal and paranasal sinus changes and secondary malignancies (Wang et al., 2000). Therefore, it is significant to develop novel approaches to improve the curative therapy and attenuate side effects of NPC.

Traditional Chinese medicine (TCM) has been used in China for more than 2000 years with approved curative therapeutic effects. People have been making efforts to understand the mechanisms of TCM’s effects as alternative medicine remedies. TCM includes many herbal medicines which proved to be effective as antitumor drugs by strengthening people’s immune system (Cho and Leung, 2007). Some of the herbs are effective in attenuating chemotherapy-induced nausea, vomiting, and peripheral neuropathy (Wu et al., 2005; Schröder et al., 2013). *Radix Ophiopogonis* (RO), the root of the perennial herbaceous plant *Ophiopogon japonicas* (validated on http://mpns.kew.org/mpns-portal/?_ga=1.111763972.1427522246.1459077346), is one of such herbs. As an important herb from Shen Nong Ben Cao Jing, it has been used in many TCM classical formulas (Li et al., 2007; Gao et al., 2012). Several studies have shown that the herb RO had been effective as an adjunctive therapy for NPC. It was reported that RO could significantly increase 1, 3, and 5 years survival rate of NPC after radiotherapy and chemotherapy (Zhang and Luo, 1994; Yuan et al., 2000). And adverse effects of NPC radiotherapy and chemotherapy were alleviated by RO-incorporated formula (Su et al., 2005; Zou et al., 2005). Moreover, by implementing RO-embedded formula, incidence of NPC distant metastasis was decreased compared with using radiotherapy and chemotherapy (Xu et al., 1989). Among 22 reported studies, RO was the most frequently used herb to treat NPC. However, the pharmacological mechanisms of RO remain unclear and it is of great importance to be elucidated (Kim et al., 2015).

Proteomics, as one of the omics-strategies, has been proved to be promising in obtaining clinical evidence and analyzing differentially expressed proteins. And the strength of proteomics has immerged as specific, cost effective, and with high-throughput yield (Janvilisri, 2015). To achieve quantification of proteins, scientists make use of liquid chromatography (LC) combined with MS. LC-MS/MS technology enables scientists to identify and quantify proteins with high efficiency. Up to date, several proteins have been identified and used as biomarkers for their significant differences in NPC patients by LC-MS/MS proteomics technologies (Cho and Leung, 2007; Xiao et al., 2007; Xiao Z.F. et al., 2010). However, the numbers of biomarkers from serum were few and mechanism behind remained unclear. In the current study, we applied a novel method-isobaric tag for relative and absolute quantification (iTRAQ) of differentially expressed proteins. The iTRAQ labeling has been proven to enhance the analytical accuracy and precision, and it has been successfully applied to establish reliable protein profiles in ibuprofen treated neuron cells, atenolol treated vascular smooth muscle cells and HepG2 cells transfected with HBV genome (Ross et al., 2004; Sui et al., 2008; Zhang et al., 2008, 2009). By using iTRAQ-coupled 2-dimensional LC-MS/MS analysis, we identified13 novel proteins differentially expressed in NPC patients’ sera. These up-regulated and down-regulated proteins in NPC patients provide us new evidence to understand the mechanism behind.

Systematic approach has been recognized as one of the promising methods to achieve a holistic view of cellular events. Based on the data from literature reports, database, and experiments, systematic approaches such as network pharmacology, system biology, and network biology perform well to analyze multi-ingredient medicine, especially, for TCM herbs containing various compounds, they work quite efficiently (Li et al., 2010; Wang et al., 2012; Li and Zhang, 2013). This property of systematic approach allows us to gradually elucidate and understand the mechanism of multi-compound medicine treatments.

**FIGURE 1:**
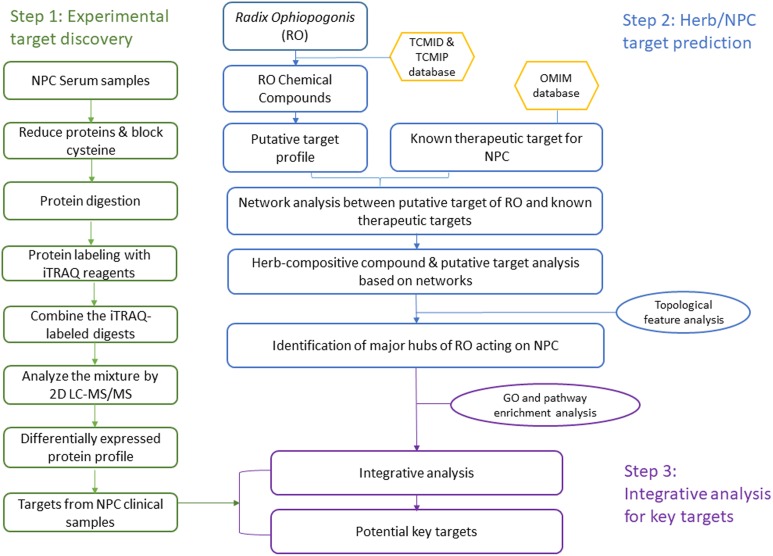
A systematic workflow of the integrative approach for deciphering the pharmaceutical mechanism of the herb *Radix Ophiopogonis* on NPC.

In this study, we developed an integrative approach to understand the TCM herb RO’s effects on NPC by linking bioinformatics prediction with clinical experiments. Our strategy includes three steps: (1) discover differentially expressed proteins in NPC clinical samples; (2) predict the RO and NPC putative targets; and (3) investigate the relationship between experimental results and bioinformatics prediction to find potential key targets with important role in the integrative network analysis. Figure 1 shows a flowchart of the whole procedure.

## Materials and Methods

### Step 1: Experimental Candidates Discovery: Differentially Expressed Protein Profile

#### Serum Samples Collection

Serum samples of NPC patients and healthy controls were collected from National Cancer Centre of Singapore. The study group was consisted of 30 patients with NPC and 10 healthy controls randomly distributed in Singapore. This study was approved by Clinical Trial Committee at National Cancer Centre of Singapore and conducted in accordance with the ethical guidelines of the Declaration of Helsinki. Serum samples were stored at −80°C prior to further experiments. The written informed consent was obtained from all the participants of this study.

**Table 1:** Differentially expressed proteins from NPC serum samples.

Biomarker candidates		Sequence coverage (%)	Protein ratio (NPC/control)	*P*-value	Peptides	Protein score
P01009	Alpha-1-antitrypsin	45.61	2.02	0.034	22	37.04
P01857	Ig gamma-1 chain C region	53.51	3.32	0.001	20	26.76
P01859	Ig gamma-2 chain C region	39.83	0.51	6.26E-08	16	12.11
P04114	Apolipoprotein B-100	3.37	1.4	0.005	10	21.8
P02763	Alpha-1-acid glycoprotein 1	29.85	2.32	3.68E-04	6	10.2
Q14624	Inter-alpha-trypsin inhibitor heavy chain H4	4.44	1.4	1.75E-04	3	7.42
P19823	Inter-alpha-trypsin inhibitor heavy chain H2	6.413	0.76	7.00E-03	3	7.55
P01008	Antithrombin III	7.83	3.34	2.59E-04	3	6.58
P05155	Plasma protease C1 inhibitor	11.66	1.54	1.62E-04	3	7.01
P01023	Alpha-2-macroglobulin	28.14	0.53	7.73E-10	27	53.11
P00738	Haptoglobin	35.22	1.39	1.20E-02	12	21.41
P02647	Apolipoprotein A-I	45.75	0.57	2.17E-08	14	25.64
P04196	Histidine-rich glycoprotein	10.52	0.74	7.97E-07	3	6.52

#### Protein Precipitation and Labeling With iTRAQ Reagents

Protein concentration of each sample was determined by 2-D Quant Kit (GE Heathcare). For each sample, serum volume (10 μl) equal to 100 μg was precipitated by adding four volumes of cold acetone. Mixtures were incubated at −20°C for 2 h. Followed by iTRAQ protocol (Applied Biosystems), proteins in sera were dissolved, denatured, and cysteine blocked. Then 20 μl of 0.25 μg/μl sequence grade modified tripsin (Promega) were used to digest each protein solution at 37°C overnight before labeling it with iTRAQ reagents.

Patients’ sera proteins were labeled individually with iTRAQ reagents 115, 116, and 117 and healthy controls were labeled with iTRAQ reagent 114. After labeling, four labeled samples containing three NPC sera and one healthy serum were mixed together into one fresh tube for further analysis. Thus 10 batches of serum proteins labeled with iTRAQ reagents were prepared for 2D LC-MS/MS analysis. The workflow of iTRAQ labeling is summarized in Figure 1.

#### Online 2-D LC-MS/MS Analysis

After proteins are digested into separate labeled peptides, samples are ready to go to LC separation. In the first dimension, 5 μl of the combined peptide mixture were injected onto a strong cation exchange column (0.32 mm × 50 mm, 5 μm). A reversed phase column was employed as second dimension and these two enrichment columns trapped the peptides. Buffer D, constituted of series of increasing concentration of KCl salt solution as 10, 20, 30, 40, 50, 60, 80, 100, 300, and 500 mM, was used to elute the retained peptides in a stepwise manner (Sui et al., 2008). Each KCl solution was used as a 100 min run. In the running process, some of the peptides bound to the SCX column during the flow. Peptides passing SCX column were trapped in the ZORBAX 300SB-C18 enrichment column I (0.3 mm × 5 mm, 5 μm) and washed by buffer A (5% acetonitrile, 0.1% formic acid) at rate of 0.5 ml/min to remove the excess reagents. Two enrichment columns were alternatively switched into the solvent path of the nanopump by a 10-port valve. The previous pathway was considered as position 1. In the following run (100 mM KCl), the 10-port switching valve was switched to position 2. A total of 5 μl of 10 mM KCl solution were injected into the SCX column to elute retained peptides to column II which was washed isocratically using the loading buffer for 100 min at 0.5 ml/min for removing excess reagent. The column I which trapped the unbound peptides in the first run was switched into the solvent path of the nanopump. Peptides were eluted using the buffer B (0.1% formic acid) and the buffer C (95% acetonitrile, 0.1% formic acid) with a nanoflow gradient starting with 5% of the buffer C and increasing up to 80% of the same buffer C over 100 min at a flow rate of 500 nL/min. This gradient wash is to make sure most of the peptides can be eluted out, and further separation was achieved in analytical Zorbax 300SB C-18 reversed-phase column (75 μm × 50 mm, 3.5 μm), which separated peptides according to their hydrophobic-hydrophilic properties.

The LC was used to separate peptides and MS to identify protein by its unique *m/z* ratio. In the MS detection, the HP1200 LC system (Agilent Technologies) was interfaced with a QSTAR XL (Applied Biosystems-MDS Sciex) mass spectrometry. The ionization method used was electrospray ionization. Survey scans were acquired from *m*/*z* 300–1500 with up to two precursors selected for MS/MS from *m*/*z* 100–2000 using dynamic exclusion, and the rolling collision energy was used to promote fragmentation. Finally, detection was performed in software for every 100-min run coming from LC.

#### Data Analysis and Interpretation

Due to unique *m/z* ratio, peptide identifications were performed using ProteinPilot^TM^ Software 2.0 packages (Applied Biosystems, Software Revision 50816). Each MS/MS spectrum was searched within the Uniprot protein database, and proteins were accepted if ProtScore value was more than 2.0, which gave the confidence value of 99%. The database allowed for iTRAQ reagent labels at N-terminal residues, internal K and Y residues, and the methylmethanethiosulfate-labeled cysteine as fixed modification, plus one missed cleavage. The analysis for the iTRAQ experiments was performed with ProteinPilot 2.0. The cutoff for the confidence settings was 75, and the tolerance settings for peptide identification in ProteinPilot searches were 0.15 Da for MS and 0.1 Da for MS/MS. ProteinPilot pooled data from all the series of runs of increasing concentration of KCL in one experiment. All identifications were manually inspected to minimize machine-related errors. Relative quantification of proteins in the case of iTRAQ was performed on the MS/MS scans and was the ratio of the areas under the peaks at 114, 115, 116, and 117 Da which were the masses of the tags that correspond to the iTRAQ reagents. The relative amount of a peptide in each sample was calculated by dividing the peak areas observed at 115, 116, and 117 *m*/*z* by that observed at 114 *m*/*z*. The calculated peak area ratios were corrected for overlapping isotopic contributions, and were used to estimate the relative abundances of a particular peptide. For proteins with two or more qualified peptide matches, three average peak area ratios (designated as 115/114, 116/114, and 117/114) were calculated using the peak area ratios of the peptides originating from the same protein. To account for small differences in protein loading, these ratios have been normalized using the overall ratios for all proteins in the sample, as recommended by Applied Biosystems. In the study, protein quantification data with relative expression of >1.2 or <0.8 were considered as significant difference from control.

#### Statistical Analysis

We consider these proteins as potential biomarkers for further analysis under the following criteria: Unused protein score was more than two (above 99% confidence); At least two peptides were identified to achieve a high confidence (>99%); and Student’s *t*-tests were employed and acceptable *p*-values for biomarkers (<0.05) fulfill the quantification requirement.

### Step 2: RO Target Prediction and NPC Known Therapeutic Target Collection

#### Compositive Compounds of *Radix Ophiopogonis*

The chemical compounds of RO were obtained from TCM Database@Taiwan, China (Chen, 2011)^1^ and TCMIP database (Huang et al., 2015; Haiyu et al., 2016; Yu et al., 2017). We collected 20 chemical compounds contained in RO under the criteria “Tanimoto scores > 0.8.” The score represents the similarity degree of the certain chemical components to that of known drugs. Tanimoto score ranges from 0–1, where “0” means the completely different structures between ingredients and known drugs, and “1” means the same structures of two compounds. This Tanimoto score is the criteria to remove redundant compounds. The known compounds with high structural similarity (the structural similarity score is higher than 0.8) were identified as the putative compounds of the herb RO. The compounds with similarity score lower than 0.8 were seen as redundant compounds. Details about chemical compounds contained in RO are provided in Supplementary Table S1.

#### Prediction of RO Putative Targets

We use Traditional Chinese Medicines Integrated Database^2^ and TCMIP^3^ using MedChem Studio (version 3.0) to identify the known putative target of RO (Xue et al., 2013; Xu H.-Y. et al., 2018; Xu S. et al., 2018). As described above using Tanimoto scores to select RO putative targets, after removing redundancy, we found 240 RO putative gene targets. These targets are also provided in Supplementary Table S1.

#### Known Therapeutic Targets of NPC Treatments

Known therapeutic targets for the treatment of NPC were collected from two databases: Drugbank database ^4^ (Wishart et al., 2008) and the OMIM database ^5^ (Hamosh et al., 2005). Only Food and Drug Administration (FDA) approved drugs used in human were chosen. In total, 134 therapeutic targets were selected as NPC-related targets. Detailed information on the collected known therapeutic targets is provided in Supplementary Table S2.

#### Pathway Enrichment Analysis of RO and NPC Putative Targets

Both RO putative targets and known therapeutic targets of NPC treatments were analyzed through pathway enrichment analysis. It was performed using the Database for Annotation, Visualization and Integrated Discovery database (DAVID^6^, version 6.8) and Kyoto Encyclopedia of Genes and Genomes (KEGG)^7^ database (Kanehisa and Goto, 2000; Dennis et al., 2003). We did the pathway enrichment analysis in two steps. First, we input RO putative targets and known NPC targets into DAVID database and KEGG database. The pathways are outputs of these gene input. Second, certain pathways were analyzed and chosen due to their physiological and pharmaceutical importance.

#### Network Analysis Between RO Putative Targets and NPC Known Therapeutic Targets

The interaction data of RO putative targets and NPC known therapeutic targets were derived from STRING (Search Tool for the Retrieval of Interacting Genes/Proteins^8^, vision 10.5) database. Interaction confidence of proteins was indicated by scores. We chose the targets as major hubs when the combined score of those targets was higher than the medium value. Detailed information obtained is provided in Supplementary Table S3. Then we used Cytoscape (Vision 3.5.1, Boston, MA, United States) to visualize the networks.

#### Identification of Major Predicted Hubs Based on Above Interaction Network

Based on the above network, we further identified the major nodes in the direct protein-protein interactions. We employed the three topological features “degree,” “betweenness,” and “closeness” as criteria to screen the putative targets for topological importance. “Degree” was defined as the number of links to one node. “Node betweenness” was defined as the number of the shortest paths between pairs of nodes that ran through one node. “Closeness” was defined as the inverse of the farness, which was the sum of the node distances to all other nodes. The closeness centrality can be regarded as a measure of how long it will take to sequentially spread information from the node to all the other nodes. Degree, node betweenness, and closeness centralities can be used to measure a protein’s topological importance in the network. The larger a protein’s degree/node betweenness/closeness centrality, the more important that protein is in the network (Wang et al., 2012). Every node has its unique topological features as described above. We calculated the median value of those topological values. Nodes with their topological values higher than the average were chosen as major hubs. After screening with above criteria, 12 candidates were identified as major predicted hubs, provided in Supplementary Table S4.

### Step 3: Identification of Key Targets of RO Against NPC

#### Interactive Analysis for Major Predicted Hubs and Experimental Candidates

As mentioned in step 1, major predicted hubs and differentially expressed proteins as experimental candidates were input as major nodes into STRING database. Detailed information obtained is provided in Supplementary Table S5. Then we used Cytoscape (Vision 3.5.1, Boston, MA, United States) to identify and visualize the key targets within networks.

#### Pathway Enrichment Analysis of Predicted and Experimental Targets

In order to elucidate the mechanism of herb-NPC interactions with respect to experimental evidence, as described previously, DAVID and KEGG databases were used for pathway enrichment analysis. Additionally, we used Protein ANalysis THrough Evolutionary Relationships Classification System (PANTHER^9^, vision 13.1, updated on Feb 03, 2018) to do the statistical overrepresentation test to further expect the key targets (Thomas et al., 2006; Mi et al., 2013, 2017). Based on the pathway enrichment analysis, potential key targets were proposed due to their important role in the network and their functions.

## Results

**FIGURE 2:**
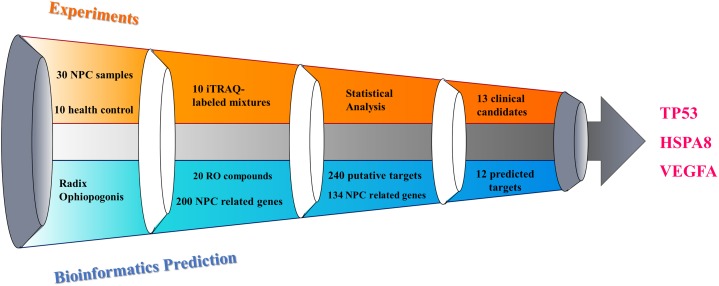
Illustration of key targets discovery process for deciphering the pharmacological mechanism of the herb RO in the treatment of NPC.

The process of discovering the key targets of the herb RO in the treatment of NPC, using an integrative approach with respect to ITRAQ-coupled 2-D LC-MS/MS analysis of differentially expressed serum proteins, is elucidated in Figure 1. The results and work flow was shown in Figure 2.

**Table 2:** Major GO biological pathways and KEGG pathways shared by major predicted and experimental targets.

Term	Candidates	*P*-value
**Biological processes**		
GO:0000122 negative regulation of transcription from RNA polymerase II promoter	SMAD4, TP53, CTNNB1	0.0236
GO:0005719 nuclear euchromatin	CREB1, CTNNB1	0.0282
GO:0008285 negative regulation of cell proliferation	SMAD4, HRG, CTNNB1	0.0236
GO:0032956 regulation of actin cytoskeleton organization	HRG, ABL1	0.0279
GO:0050821 protein stabilization	APOA1, CREB1	0.0054
**KEGG pathways**		
ptr05205:proteoglycans in cancer	AKT1, VEGFA, TP53, CTNNB1	0.0101
ptr04014:Ras signaling pathway	AKT1, VEGFA, ABL1	0.0118
ptr04015:Rap1 signaling pathway	AKT1, ADCY1, VEGFA, CTNNB1	0.0029
ptr04022:cGMP-PKG signaling pathway	AKT1, ADCY1, CREB1	0.0223
ptr04110:cell cycle	SMAD4, TP53, ABL1	0.0328
ptr04151:PI3K-Akt signaling pathway	AKT1, CREB1, VEGFA, TP53	0.0107
ptr04152:AMPK signaling pathway	AKT1, CREB1, EEF2	0.0403
ptr04310:Wnt signaling pathway	SMAD4, TP53, CTNNB1	0.0393
ptr04510:focal adhesion	AKT1, VEGFA, CTNNB1	0.0342
ptr04919:thyroid hormone signaling pathway	AKT1, TP53, CTNNB1	0.0277
ptr05169:Epstein–Barr virus infection	AKT1, TP53, HSPA8	0.0292

### Part I: Experimental Targets Discovery

#### Identification of NPC Differentially Expressed Proteins

According to LC-MS/MS analysis and ProteinPilot 2.0 scanning, each serum sample showed unique protein profile. Among these proteins, 13 proteins were selected as potential biomarkers for future NPC management based on the fact that more than 25 out of 30 patients shared a similar protein profile. And these proteins’ relative concentration was either >1.2 or <0.8, showing significant difference compared with healthy subjects. Compared with the reported data, most of the proteins identified in our study were unique in their cellular functions.

**FIGURE 3:**
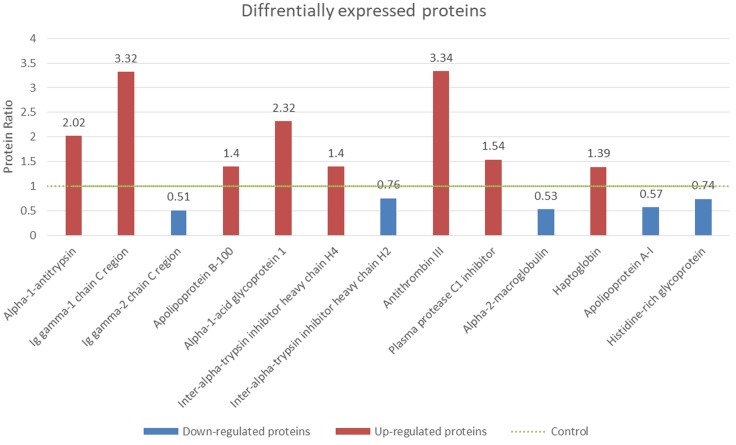
Identification of differentially expressed proteins from NPC serum samples. The protein concentrations of controls were normalized to 1 (green dotted line). The fold changes of the NPC patients’ serum and healthy control were shown. Red columns (>1.2 fold) were identified as up-regulated proteins and blue columns (<0.8 fold) were down-regulated proteins (*p* < 0.05).

Under the criteria mentioned, nine proteins were identified as up-regulated biomarkers for their relatively higher concentration (>1.2 fold) in NPC patients compared with healthy controls, while four proteins were identified as down-regulated biomarkers. Both up-regulated and down-regulated biomarkers were summarized and shown with sequence coverage, average concentration changing fold, number of distinct peptides detected and protein score (Table 1 and Figure 3).

#### MS/MS Spectra of Representative Peptides

**FIGURE 4:**
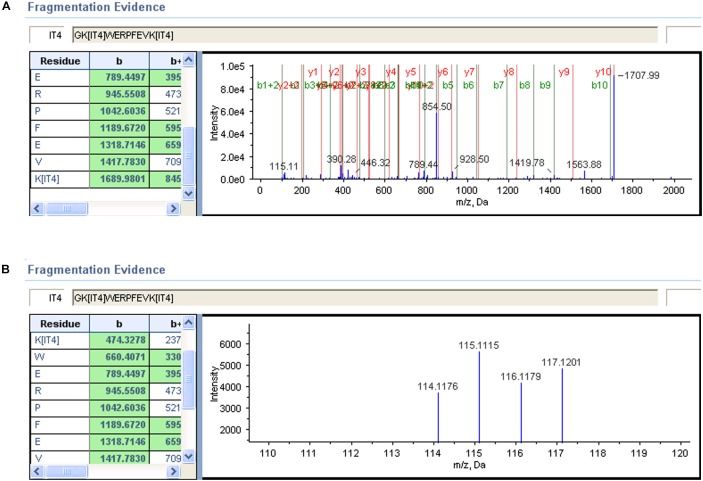
Alpha-1-antitrypsin MS/MS spectrum of a representative peptide. **(A)** A representative peptide’s mass spectrum of this biomarker. Each peak represented one residue by detecting its unique *m/z* ratio. **(B)** The signal intensity of iTRAQ labeled residues from 114 to 117.

To elucidate the process of biomarker’s identification, two representative MS/MS spectra were selected (Figure 4). The high confidence peptides were displayed in one dimension in one spectrum. The other spectrum provided a zoom-in picture, showing different performance of iTRAQ labeled serum samples. The NPC patients were labeled with iTRAQ reagents from 115 to 117, while healthy control was labeled with reagent 114. The whole protein concentrations were calculated after summarizing all detected spectra information under provided criteria.

### Part II: Bioinformatics Prediction

#### Chemical Information About RO and the Prediction of Its Potential Targets

Following the procedure descripted previously using TCM Database@Taiwan and TCMIP database, we collected 20 chemical compounds contained in RO, included in Supplementary Table S1. Traditional Chinese Medicines Integrated Database was used to identify the putative targets of RO-compositive compounds. As a result, we found 240 putative gene targets which are also included in Supplementary Table S1.

#### RO Compositive Compound-Putative Target Network

**FIGURE 5:**
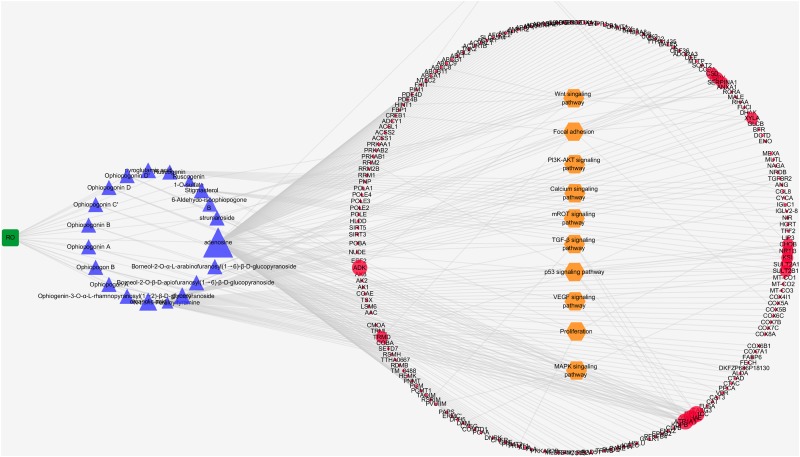
Interaction network between compositive compounds of RO and their putative targets which was built and visualized with Cytoscape. Edges: interactions between compositive compounds of RO and their putative targets; green square node: herb *Radix Ophiopogonis*(RO); blue triangular nodes: compositive compounds of RO; red round nodes: putative targets of compositive compounds of RO; orange hexagonal nodes: the main pathways from enrichment analysis of major targets. The size of nodes indicates the strength of interactions.

In order to understand the RO’s interaction with its putative targets, we first built up the herb compositive compound-putative target network. The network was consisted of 224 nodes (one herb, 20 compositive compounds, and 240 putative targets) and 263 edges. The mean number of putative targets per compositive compound was 2.295. According to the degree (the number of links to one node) of nodes, larger nodes indicated strong interaction with other compounds and targets, as shown in Figure 5. By pathway enrichment analysis, we found that these RO putative targets were frequently involved in 11 pathways and biological processes including Wnt signaling pathway, cAMP signaling pathway, focal adhesion, PI3K-AKT signaling pathway, calcium signaling pathway, mROT signaling pathway, TGF-β signaling pathway, p53 signaling pathway, VEGF signaling pathway, proliferation, and MAPK signaling pathway.

#### RO Compositive Compound-Putative Known NPC Target Network

**FIGURE 6:**
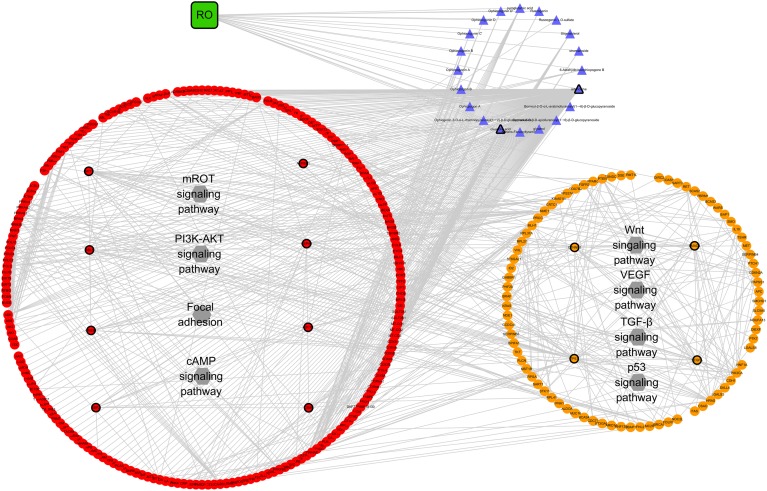
Network analysis for the interaction between RO compositive compound-putative target and known NPC targets visualized with Cytoscape. Edges: interactions among the herb RO, compositive compounds, putative targets, and known therapeutic targets for the treatment of NPC. Green square nodes: the herb RO; blue triangular nodes: compositive compounds of RO; red round nodes: putative targets of compositive compounds of RO; orange round nodes: known therapeutic targets for the treatment of NPC; gray hexagonal nodes: the main pathways from enrichment analysis of major nodes. Nodes marked with dark rings: major nodes, the “degree,” “node betweenness,” and “closeness” of which were all larger than the corresponding median values.

The RO compositive compound-putative known NPC target network was constructed to evaluate the effects of RO on NPC. The interaction data of RO putative targets and NPC known therapeutic targets were acquired as previously described in Section “Materials and Methods.” As provided in Figure 6, the network is consisted of 307 nodes (containing one ingredient, 20 compositive compounds contained in RO, 240 RO putative targets, and 134 known therapeutic of NPC) and 738 edges. As mentioned in Section “Materials and Methods,” three topological features, “degree,” “betweenness,” and “closeness,” were employed as criteria to screen the putative targets for topological importance. Only nodes, with topological features higher than the corresponding median values, were identified as major nodes. As a result, we identified 15 major nodes containing one herb, two compositive compounds contained in RO, eight putative targets, and four known therapeutic targets for the treatments of NPC. Details concerning the topological features of the 15 major nodes in this network are shown in Supplementary Table S4. Besides the herb and two compounds of RO, 12 candidates (eight RO compositive compounds and four known therapeutic NPC compounds) were considered as the major RO-NPC predicted targets. Based on pathway enrichment analysis of major nodes, mROT signaling pathway, PI3K-AKT signaling pathway, focal adhesion, and cAMP signaling pathway showed strong connection with RO targets. Meanwhile, NPC targets also indicated their frequent involvements in Wnt signaling pathway, VEGF signaling pathway, TGF-β signaling pathway, and p53 signaling pathway.

### Part III: Identification of Key Targets of RO Against NPC and Function Analysis

**FIGURE 7:**
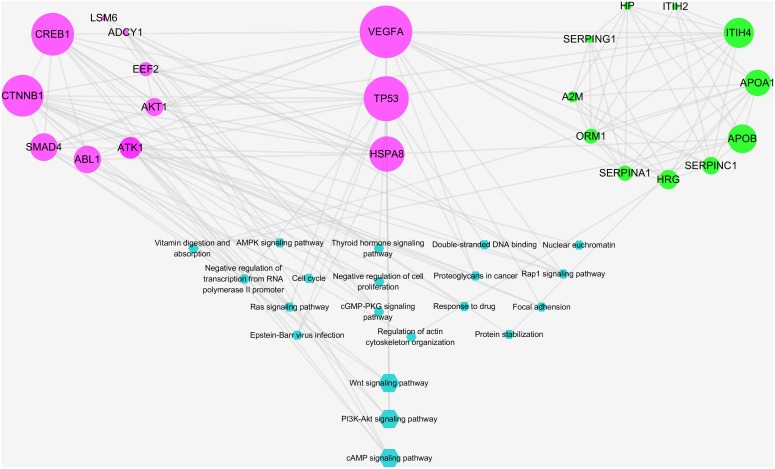
Integrative pathway enrichment analysis showing the interaction between predicted and experimental targets. Edges: interactions between predicted and experimental targets involved in their pathways; pink round nodes: major RO-NPC predicted targets; green round nodes: experimental targets of NPC; blue hexagonal nodes: the main pathways from enrichment analysis of major targets. The size of nodes indicates the strength of interactions, indicating the relative importance of VEGFA, TP53, and HSPA8. The bigger blue hexagonal nodes show PI3K-AKT signaling pathway, Wnt signaling pathway, and cAMP signaling pathway were strongly connected pathways.

A total of 12 major putative targets and 13 experimental candidates were input into STRING database in order to further understand the pharmacological mechanisms of the action of RO on NPC. As shown in Figure 7, candidates with bigger size showed stronger interactions in the network, as their topological features were examined by the criteria mentioned above. Among these targets, VEGFA, TP53, and HSPA8 were indicated as key targets because of their strong interactions in the network. Moreover, a statistical overrepresentation test run in PANTHER database was performed to expect the candidates’ possible importance. As a result, this analysis also indicated VEGFA, TP53 and HSPA8 were key targets with 32.79-fold enrichment scores (Supplementary Table S6).

According to a pathway enrichment analysis based on the GO annotation system and the KEGG pathway, the major predicated and experimental targets were frequently involved in five biological processes and 11 KEGG pathways as shown in Table 2. These biological processes and pathways include: negative regulation of transcription from RNA polymerase II promoter, nuclear euchromatin, negative regulation of cell proliferation, regulation of actin cytoskeleton organization, protein stabilization, Proteoglycans in cancer, Ras signaling pathway, Rap1 signaling pathway, cGMP-PKG signaling pathway, cell cycle, PI3K-Akt signaling pathway, AMPK signaling pathway, Wnt signaling pathway, focal adhesion, thyroid hormone signaling pathway, and Epstein–Barr virus infection. The strength of interactions between pathways and major targets was indicated by pathways’ sizes (Figure 7). As described in Figure 6, both predicted and experimental targets are involved in three significant pathways: PI3K-AKT signaling pathway, Wnt signaling pathway, and cAMP signaling pathway. Our finding suggests that key candidates VEGFA, TP53, and HSPA8 may play important role in mediating the herb RO’s action on NPC. And this integrative analysis, combing bioinformatics prediction and experimental evidences, shows potentiality in deciphering the mechanism of the herb RO in the treatment of NPC.

## Discussion

In summary, we identified 12 predicted targets (eight RO putative targets and four known therapeutic NPC targets) and 13 differentially expressed proteins as experimental targets frequently involved in five biological processes and 11 pathways. After integrative analysis incorporating predicted and experimental candidates, the discovery of VEGFA, TP53, and HSPA8 in RO’s action on NPC treatments indicates novel targets for RO in treating NPC. The integrative analysis of bioinformatics prediction and experiments can provide us novel perspective to understand the mechanism of NPC treatments in response to RO.

The iTRAQ-coupled 2-D LC-MS/MS analysis is a powerful approach to discover differentially expressed proteins. In this study, 13 proteins were found to be differentially expressed in NPC patients compared with the healthy controls. These findings provide novel clues in understanding the RO’s actions on NPC. Among the 13 biomarkers identified in our study, three pairs of proteins shared the similar structure while performing opposite regulations as NPC potential biomarkers. For example, Ig gamma-1 chain C region and Ig gamma-2 chain C region both belong to immunoglobulin with similar heavy chain. But Ig gamma-1 chain C region has higher concentration than the control and thus noted as high-level biomarker, whereas Ig gamma-2 chain C region was down-regulated. The distinct concentration of these two supposed similar biomarkers raises our interest for investigation on their structure differences. The same occurrence appeared in the other two pairs of biomarkers. Supplementary Table S7 shows three pairs of NPC biomarkers sharing similar structure but performing differently. Due to the difficulty of collecting samples, samples treated with RO were not included in this study. More experiments of treating the NPC patients with the herb RO will be employed to further understand this phenomenon.

Vascular endothelial growth factor A (VEGFA) is a growth factor active in angiogenesis, vasculogenesis, and endothelial cell growth. In this study, it showed strong relationship with Proteoglycans in cancer, Rap1 signaling pathway, PI3K-Akt signaling pathway, **focal** adhesion, and Ras signaling pathway. In these pathways, VEGFA was able to inhibit tumor angiogenesis in proteoglycans in cancer (Kerbel, 2008; Mayumi, 2008; Iozzo and Sanderson, 2011). It was reported to activate Rap1 signaling pathway, PI3K-Akt signaling pathway, Ras signaling pathway and influence **f**ocal adhesion by triggering RTK (Cross and Claesson-Welsh, 2001; Cross et al., 2003; Olsson et al., 2006; Adya et al., 2008; Claesson-Welsh and Welsh, 2013; Goel and Mercurio, 2013). Other group’s research also support VEGFA’s important role in NPC managements. Wang et al. (2010) reported polymorphism of VEGF-2578C/A associated with the risk and aggressiveness of **NPC**. Makni et al. (2016) also reported distinct association of VEGFA polymorphisms with NPC. Tian et al. (2013) found **that** VEGFA-ERK pathway was inactivated in NPC tumors treated with radiotherapy. Xu H.-Y. et al. (2018), Xu S. et al. (2018) suggested NPC vasculogenic mimicry formation was mediated by EBV-LMP1 via VEGFA/VEGFR1 signaling. These findings correlate with our study supporting that VEGFA acts a key role during NPC treatments in response to RO.

Tumor protein p53 (TP53) is a tumor suppressor in many tumor types by inducing growth arrest or apoptosis (Moshe et al., 2002). The network analysis in this study revealed that TP53 was frequently involved in proteoglycans in cancer, PI3K-Akt signaling pathway, thyroid hormone signaling pathway, Wnt signaling pathway, and Epstein–Barr virus infection. These findings relate with other groups’ reports. TP53 induces apoptosis influenced by Akt/PKB in thyroid hormone signaling pathway (Manning and Cantley, 2007; Saji and Ringel, 2010). It was also shown that TP53 could be activated by DNA damage and induce apoptosis during cell cycle (Kastan et al., 1991). In Wnt signaling pathway, TP53 is involved in proteolysis by affecting Siah-1 (Roperch et al., 1999; Kim et al., 2004). Repressed by MDM2, TP53 effects the cell survival in PI3K-Akt signaling pathway (Luo et al., 2003; Vara et al., 2004; Gang et al., 2005). It was reported that most NPC patients were infected with EBV and the virus infection was recognized as a popular factor inducing NPC development. During EBV infection, the virus makes impact on TP53 through Epstein–Barr nuclear antigen 1 (EBNA1). The identification of TP53’s role in NPC in our study was also reported by other groups. Xiao M. et al. (2010) found that genetic polymorphism of the TP53 gene was associated with risk of NPC. Hwang et al. (2009) suggested TP53 suppressed NPC tumor progression by regulating MDM2 mediated by NOLC1. Strong interactions of TP53 in the network of our study further proved its important role in cancer managements, hence, indicating the herb RO might take effects on NPC by mediating TP53.

Heat shock cognate 71 kDa protein (HSPA8) is a molecular chaperone implicated in a wide variety of cellular events. It plays a key role in the protein quality control system, ensuring the correct folding of proteins, the re-folding of misfolded proteins, and controlling the targeting of proteins for subsequent degradation (Yamamoto et al., 2010; Grove et al., 2011; Sopha et al., 2012; Goodwin et al., 2014; Li et al., 2017). The EBV infection makes the EBNA1 influence HSPA8 (Stricher et al., 2013). However, the detail information about HSPA8 involved mechanism in NPC was vague. Thus, the discovery of HSPA8 as key candidate in our study indicates that this candidate could possibly be a novel target in treating NPC. Also, it may play an exclusive role in NPC treatments by RO.

## Conclusion

This integrative pharmacology-based investigation combining iTRAQ-coupled 2-D LC-MS/MS analysis and network investigation revealed that the anti-NPC effects of RO might relate to its regulatory effects on the PI3K-AKT signaling pathway, the Wnt signaling pathway, and the cAMP signaling pathway by targeting VEGFA, TP53, and HSPA8, which could improve our understanding of NPC during deciphering the mechanism of NPC treatments in response to RO.

## Author Contributions

XF and HZ designed the research. XF performed the research and wrote the manuscript. HS, XC, and LS contributed analytic tools. MW and FZ analyzed the data.

## Conflict of Interest Statement

The authors declare that the research was conducted in the absence of any commercial or financial relationships that could be construed as a potential conflict of interest.

## Footnotes

1 http://tcm.cmu.edu.tw/, updated on 2014-02-28

2 http://www.nrc.ac.cn:9090/ETCM

3 http://www.tcmip.cn

4 www.drugbank.ca/, v3.0

5 www.omim.org, last updated October 31, 2013

6 http://david.abcc.ncifcrf.gov/home.jsp

7 http://www.genome.jp/kegg/, updated on April 18, 2016

8 https://string-db.org/

9 http://pantherdb.org/

## Supplementary Material

TABLE S1 Chemical compounds of RO and their gene targets in response to NPC. Each compound was provided with its corresponding NPC gene targets. We only select those compounds with similarity score > 0.8.

TABLE S2 Known Therapeutic Targets of NPC Treatments. The data was collected from the database Drug Bank and OMIM. Each known the rapeutic targets of NPC treatments were provided with its gene ID and full name of the protein.

TABLE S3 The interaction data of RO putative targets and NPC known therapeutic targets derived from STRING database. The node 1 and node 2 are RO putative targets and NPC known therapeutic targets. Please refer to STRING database for parameter explainations.

TABLE S4 Major Predicted Hubs from interative analysis. Major predicted hubs were selected due to their topological importance. “degree,” “betweenness,” and “closeness” as criteria to screen the putative targets for topological importance. “Degree” was defined as the number of links to one node. “Node betweenness” was defined as the number of the shortest paths between pairs of nodes that ran through one node. “Closeness” was defined as the inverse of the farness, which was the sum of the node distances to all other nodes. The closeness centrality can be regarded as a measure of how long it will take to sequentially spread information from the node to all the other nodes.

TABLE S5 Major Predicted Hubs and Experimental Candidates. The node 1 and 2 are major predicted hubs and experimental candidates. These nodes and their parameters were ready to input into STRING database.

TABLE S6 Statistical overrepresentation test from PANTHER database. The candidates’ relative importance was calculated using PANTHER database. Detail information about the parameters were discribed in the PANTHER database (http://www.pantherdb.org/).

TABLE S7 NPC differentially expressed proteins sharing similar structures but performing in opposite direction. At each line, the two biomarkers are highly related with similar structure. However, the expression of them are opposite. In NPC patients, these 3 pairs of biomarkers were expressed in the opposite direction.
