# Supplementary material for: Deciphering the Pharmacological Mechanism of the Herb Radix Ophiopogonis in the Treatment of Nasopharyngeal Carcinoma by Integrating iTRAQ-Coupled 2-D LC-MS/MS Analysis and Network Investigation
Source: Front Pharmacol. 2019 Mar 18;10:253. doi: 10.3389/fphar.2019.00253 (PMC6431671; doi:10.3389/fphar.2019.00253)
Supplement: TABLE S7 — NPC differentially expressed proteins sharing similar structures but performing in opposite direction. At each line, the two biomarkers are highly related with similar structure. However, the expression of them are opposite. In NPC patients, these 3 pairs of biomarkers were expressed in the opposite direction. [file Table_7.DOCX]

**Table III.** NPC differentially expressed proteins sharing similar structures but performing in opposite direction

| Up-regulated NPC Proteins | Down-regulated NPC Proteins |
| --- | --- |
| Ig gamma-1 chain C region | Ig gamma-2 chain C region |
| Inter-alpha-trypsin inhibitor heavy chain H4 | Inter-alpha-trypsin inhibitor heavy chain H2 |
| Apolipoprotein B-100 | Apolipoprotein A-I |
